# Editorial: Clinical management of older persons with cancer: current status and future directions

**DOI:** 10.3389/fmed.2025.1631044

**Published:** 2025-06-05

**Authors:** Aziz Karaoglu, Gülistan Bahat

**Affiliations:** ^1^Division of Medical Oncology, Department of Internal Medicine, Dokuz Eylul University, Izmir, Türkiye; ^2^Division of Geriatrics, Department of Internal Medicine, Istanbul Faculty of Medicine, Istanbul University, Istanbul, Türkiye

**Keywords:** cancer, older, management, geriatric assessment, cancer care

Globally, populations are aging. Cancer is essentially a disease of old age, and the number of older patients with cancer will increase with the aging of the population (1). Cancer management in older patients presents unique challenges that extend beyond traditional oncology paradigms. A comprehensive approach that incorporates the principles of geriatric oncology is crucial to improving both treatment outcomes and quality of life in this population. Furthermore, there is a need for more clinical trials that focus on older patients with cancer and new clinical trial designs that incorporate geriatric oncology concepts (new endpoints, expansion cohorts, frailty classifications, etc.). This Research Topic aims to present research focused on developing effective care strategies in the clinical management of older adults with cancer.

Chronological age, the Eastern Cooperative Oncology Group (ECOG) performance status, and the Karnofsky performance scale do not adequately reflect the functional diversity of older patients with cancer (2). Therefore, leading cancer organizations such as the International Society of Geriatric Oncology (SIOG), the American Society of Clinical Oncology (ASCO), the European Society For Medical Oncology (ESMO), and the National Comprehensive Cancer Network (NCCN) recommend geriatric assessment. The Comprehensive Geriatric Assessment (CGA) is the gold standard for assessing this patient population (3). However, CGA is not suitable for routine use in all older patients with cancer. Geriatric screening tools such as the Geriatrics 8 (G8), the Vulnerable Elders Survey-13 (VES-13), and Practical Geriatric Assessment (PGA) have been developed (4–6). In this Research Topic, De Schrevel et al. report that the Edmonton Frailty Scale reliably predicts CGA-identified frailty and one-year mortality in older cancer patients pre-selected as potentially frail by the G8. Since frailty is a known risk factor for poor outcomes, including increased mortality, identification of frail patients is crucial for the development of treatment plans. Sarcopenia in cancer patients has received increasing attention due to its high prevalence and association with adverse outcomes (7, 8).

Sarcopenia is an independent prognostic factor for complications and survival following surgical resection of malignancy (9). Increasing evidence shows that sarcopenia is related to the risk of adverse postoperative outcomes, including morbidity, prolonged length of hospital stay, and mortality. The study by Tirnova et al. underscores the association between low skeletal muscle mass (-as a proxy marker of sarcopenia) and increased risk of postoperative complications in older patients undergoing colon cancer surgery. This finding emphasizes the need for preoperative assessments that evaluate nutritional status and muscle mass to predict surgical outcomes.

Inadequate nutrition in older adults with cancer can reduce treatment tolerance and lead to poor treatment outcomes (10, 11). A significant relationship has been demonstrated between low prognostic nutritional index (PNI) and poor overall survival (OS) in bladder cancer. Balçik et al. demonstrated the prognostic value of the Geriatric Nutritional Risk Index (GNRI), Controlling Nutritional Status (CONUT) score, and Prognostic Nutritional Index (PNI) in patients with bladder cancer. They reported that both GNRI and CONUT scores may serve as useful predictors of survival in metastatic bladder cancer patients over 70 years of age.

Breast cancer is a heterogeneous disease, with patients who have similar prognostic features experiencing diverse outcomes. This highlights the need for further research on new prognostic factors, particularly for older patients. Yu et al. proposed a dynamic-effect Restricted Mean Time Lost (RMTL) regression model to investigate the time-varying effects of prognostic factors in the context of competing risk survival data. This study is the first to consider both competing risks and time-varying effects, with the real-time effects differing from previous one-tailed analyses. Applying this model to an older early-stage breast cancer cohort, they showed that protective factors like positive estrogen receptor status and chemotherapy lost impact over time, while the benefit of breast-conserving surgery and the negative effects of advanced tumor stage and grade increased. This new framework may support personalized decision making by reflecting how risks change over time and may provide clinicians with a more accurate and personalized understanding of prognosis in the geriatric oncology setting.

Telemedicine is playing an increasingly important role in geriatric oncology (12). The Cancer and Aging Interdisciplinary Team (CAIT) clinic at Memorial Sloan Kettering Cancer Center presents the findings of a study examining the role of telemedicine in older adults undergoing cancer treatment (Alexander et al.). They found that 77% of 288 patients (aged 67–100) preferred telemedicine visits. Factors such as advanced age, lower educational level, abnormal cognitive screening results, impaired performance status, instrumental activities of daily living (IADL) dependency, and poor social support were associated with preferring in-person visits. The study emphasizes the significant potential of telemedicine to optimize cancer care in older adults, improve access to care, reduce the burden of in-person visits, and enhance quality of life.

Supportive care provides psychological and social support, helping older patients with cancer cope with the emotional and social challenges they encounter during treatment. Lian et al. examined the changes in supportive care needs, quality of life, and social support among older patients with colorectal cancer undergoing chemotherapy. In this longitudinal study, 155 patients were followed over multiple chemotherapy cycles, with the results showing a significant increase in the need for supportive care, particularly psychological support and patient care, as treatment progressed. Meanwhile, the patients' quality of life and social support gradually deteriorated throughout the chemotherapy cycles.

In older adults with cancer, suicide risk is a critically important concern, with studies demonstrating a higher incidence of suicide in this group (13). Older patients with prostate cancer have also been shown to be at elevated risk for suicidal ideation. In their study, Yang et al. developed a predictive model for suicide risk in prostate cancer survivors using the Surveillance, Epidemiology, and End Results (SEER) data from over 238,000 patients. Their model, based on seven accessible clinical variables (age, race, marital status, income, prostatic specific antigen (PSA) levels, metastatic stage, and surgical status), demonstrated good predictive accuracy and identified high-risk individuals as having a 3.5-fold greater suicide risk than their low-risk counterparts. These findings underscore the importance of integrating routine psychosocial screening and psycho-oncological interventions into the care of older patients with prostate cancer to mitigate suicide risk and support overall wellbeing.

The articles in this Research Topic reflect the growing body of knowledge and evolving clinical paradigms in geriatric oncology. Optimizing care for older adults with cancer requires ongoing advancements in education, clinical research, and healthcare systems. Integrating geriatric principles into oncology training, designing inclusive clinical trials for older and frail patients, and implementing geriatric assessment are essential steps. Additionally, supportive health policy frameworks, including reimbursement models, are needed. Collaborative, interdisciplinary efforts are essential for older cancer patients.
